# Personality disorders and Axis I comorbidity in adolescent outpatients with ADHD

**DOI:** 10.1186/s12888-016-0871-0

**Published:** 2016-06-01

**Authors:** Hans Ole Korsgaard, Svenn Torgersen, Tore Wentzel-Larsen, Randi Ulberg

**Affiliations:** Department for Child and Adolescent Mental Health (The Nic Waal Institute), Lovisenberg Diakonale Hospital, P.O. Box 4970, Nydalen, N-0440 Oslo, Norway; Centre for Child and Adolescent Mental Health, Eastern and Southern Norway, Oslo, Norway; Department of Psychology, University of Oslo, Oslo, Norway; Norwegian Centre for Violence and Traumatic Stress Studies, Oslo, Norway; Vestfold Hospital Trust, Tønsberg, Norway; Institute of Clinical Medicine, University of Oslo, Oslo, Norway

**Keywords:** ADHD, Axis I, Comorbidity, Conduct disorder, Personality disorder, Adolescent, Outpatient

## Abstract

**Background:**

Attention deficit hyperactivity disorder (ADHD) is a lifelong condition which carries great cost to society and has an extensive comorbidity. It has been assumed that ADHD is 2 to 5 times more frequent in boys than in girls. Several studies have suggested developmental trajectories that link ADHD and certain personality disorders. The present study investigated the prevalence of ADHD, common Axis I disorders, and their gender differences in a sample of adolescent outpatients. We also wanted to investigate the relationship between ADHD and personality disorders (PDs), as well as how this relationship was influenced by adjustment for Axis I disorders, age and gender.

**Methods:**

We used a sample consisting of 153 adolescents, aged 14 to 17 years, who were referred to a non-specialized mental health outpatient clinic with a defined catchment area. ADHD, conduct disorder (CD) and other Axis I conditions were assessed using the Mini International Neuropsychiatric Interview (MINI). PDs were assessed using the Structured Interview for DSM-IV Personality (SIDP-IV).

**Results:**

13.7 % of the adolescents met diagnostic criteria for ADHD, with no significant gender difference. 21.6 % had at least one PD, 17.6 % had CD, and 4.6 % had both ADHD and a PD. There was a significantly elevated number of PD symptoms in adolescents with an ADHD diagnosis (*p* = 0.001), and this relationship was not significantly weakened when adjusted for age, gender and other Axis I disorders (*p* = 0.026). Antisocial (*χ*^2^ = 21.18, *p* = 0.002) and borderline (*χ*^2^ = 6.15, *p* = 0.042) PDs were significantly more frequent in girls than in boys with ADHD.

**Conclusions:**

We found no significant gender difference in the prevalence of ADHD in a sample of adolescents referred to a general mental health outpatient clinic. Adolescent girls with ADHD had more PDs than boys, with antisocial and borderline PDs significantly different. The present study suggests that ADHD in girls in a general outpatient population may be more prevalent than previously assumed. It especially highlights the importance of assessing antisocial and borderline personality pathology in adolescent girls presenting with ADHD symptoms.

## Background

### ADHD and personality disorders

Attention deficit hyperactivity disorder (ADHD) is a common and often lifelong condition which carries great cost to society and has an extensive psychiatric comorbidity [1–4]. It manifests during early childhood, previous to other Axis I diagnoses, and is associated with a broad range of other health-related issues, such as impulsive behaviors, greater number of traumas, lower quality of life, reduced social functioning, and homelessness, even after adjusting for additional comorbidity [5, 6].

The worldwide prevalence of ADHD has been estimated at about 3–5 % [7, 8], but one study reported a prevalence of 8.5 % [9]. ADHD may be more prevalent than previously assumed [10]. A recent study suggested that the prevalence of ADHD may be increasing, but this could also be due to increased clinical alertness and improved diagnostic procedures [11].

ADHD is generally considered to be more prevalent in boys than in girls, with male/female ratio estimates ranging from 2:1 to 9:1 [8, 12]. However, ADHD may be experienced by larger numbers of females than has previously been considered [10].

ADHD has been associated with anxiety, mood, and disruptive behavioral disorders [9]. In a sample of twins and siblings no significant gender differences in comorbidity for externalizing disorders were found [13]. In a five-year follow-up study of a cohort of children with ADHD, 68.9 % continued to meet full criteria for ADHD, exhibiting high levels of antisocial behavior, criminal activity and substance use problems [14].

In DSM-IV and DSM-5, personality disorder (PD) categories may be applied to adolescents when the individual’s particular maladaptive personality traits are pervasive, persistent, and unlikely to be limited to a particular developmental state or an episode of an Axis I disorder. With the exception of antisocial PD (ASPD), any PD can be diagnosed in a person under 18 years of age if the diagnostic features have been present for at least 1 year [15, 16]. However, in studies on PDs in adolescence, the DSM-IV age criterion for ASPD is waived [17–19].

PDs are common, with adult prevalence numbers of 10–15 % in the general population [20], up to 40 % in outpatient samples, and up to 71 % in inpatient samples [21]. In adolescents, prevalences range from 6 to 17 % in community samples, and in clinical samples from 41 to 64 % [18, 19].

Research supports the assumption that PD symptoms emerge at an early age and are related to health-risk behaviors in adolescence as well as young adulthood [22–24], but PDs may have a better prognosis than previously assumed. Maladaptive personality traits may change in severity or expression over time; still they often lead to persistent functional impairment and reduced quality of life even if the diagnostic threshold for a specific PD is no longer reached [25, 26].

Borderline PD (BPD) has a lifetime prevalence of 2.7 % in the general population; it seems to be equally prevalent among men and women [27]. Diagnosing BPD in young persons can be challenging [28], but there is an increasing awareness of predisposing factors and adolescent presentation of BPD [29–33]. Recent work has demonstrated that BPD is as reliable and valid in adolescents as in adults [32, 34, 35]. One study suggested that late-latency children are about half as likely as adults to meet DSM-IV criteria for BPD [36].

Few studies have reported on gender differences [18] and gender might not play a defining role in symptom expression [36].

### ADHD, PDs, and Axis I comorbidity

The question has been posed if ADHD can be considered an early stage in the development of BPD. A comprehensive literature review found data that provide a basis for the hypotheses that ADHD is either an early developmental stage of BPD, or that the two disorders share an environmental and genetic aetiology [37].

Adults with severe BPD frequently show a history of childhood ADHD symptoms. Persisting ADHD correlates with the frequency of co-occurring Axis I and PDs [38–41]; for example, the presence of ADHD tends to make BPD more disruptive [42]. A study of treatment refractory adolescents and young adults found unrecognized ADHD in 6 % of the patients [43].

In prisoners childhood and adult ADHD symptoms were found to be positively correlated with BPD and negatively correlated with compulsive personality pathology. Axis I disorders were not significantly related to childhood ADHD [44]. A study on probationers with BPD reported substantially more symptoms of ADHD, anxiety and depression compared to subjects without BPD [45].

Several studies have suggested developmental trajectories that link ADHD, bipolar disorder and certain PDs, especially BPD. The exact nature of these aetiological links is not known [41, 46], but mood lability has been suggested as a common denominator [47].

Speranza and colleagues found comorbid ADHD to influence the clinical presentation of adolescents with BPD, and that comorbid ADHD was associated with higher rates of disruptive disorders, with a trend towards a greater likelihood of cluster B PDs and with higher levels of impulsivity, especially of the attentional/cognitive type [42]. Prada and colleagues found that ADHD and BPD-ADHD patients show a higher level of impulsivity than BPD and control subjects [48].

Individuals diagnosed with childhood ADHD were found to be at increased risk for PDs in late adolescence, specifically borderline (OR = 13.16), antisocial (OR = 3.03), avoidant (OR = 9.77), and narcissistic (OR = 8.69) PDs. Those with persistent ADHD were at higher risk for antisocial (OR = 5.26) and paranoid (OR = 8.47) PDs but not the other PDs, when compared to those in whom ADHD remitted. These results suggest that ADHD portends risk for adult PDs, but that the risk is neither uniform across disorders, nor uniformly related to child or adult diagnostic status [49].

Females with ADHD and BPD seem to share more clinical features than males [50, 51]; in adult outpatients a significant association between retrospectively assessed ADHD symptoms and current BPD features was found only in the female subsample [52].

### Aims

The objective of the present study, performed on a clinical sample of consecutively referred adolescent outpatients, was to

1. Investigate the prevalence of ADHD and common Axis I disorders, including possible gender differences.
2. Investigate the relationship between ADHD and PDs. We also wanted to assess the influence of adjusting for Axis I disorders, age and gender on the relationship between ADHD and PDs.

## Methods

### Participants

The sample consisted of adolescents aged 14–17 years who were referred to a mental health outpatient clinic for children and adolescents in Oslo (The Nic Waal Institute, Lovisenberg Diakonale Hospital). The Nic Waal Institute is serving four city districts with a population of mixed socioeconomic status, representing all social classes including immigrant workers and well-educated middle and upper class families. The catchment area comprises a total population of 25, 000 children and adolescents from 0 to 17 years of age.

Study inclusion took place from February 2005 to April 2007. Exclusion criteria were the need for immediate hospitalization or other urgent therapeutic measures, clinically assessed mental retardation, lack of fluency in the Norwegian language, and absence of the evaluator at the time of referral.

### Measures

#### ADHD

A primary screening for ADHD was performed using the six-item Adult ADHD Self-Report Scale Screener version 1.1 (ASRS Screener) in a Norwegian translation [53]. The ASRS Screener is derived from the 18-item ASRS 1.1 Symptom Checklist [54] and is designed to screen for and estimate the prevalence of ADHD in community samples, as well as in population surveys and at an individual level. The measure is reliable and valid in clinical settings [55] and has repeatedly been shown to be in strong concordance with clinician diagnoses [56].

If the primary screening with the ASRS Screener was positive, the Mini International Neuropsychiatric Interview-PLUS (MINI-PLUS) section W (ADHD in children/adolescents) was used as a diagnostic test instrument [57] for a final diagnosis of ADHD.

### Axis I disorders

The Mini International Neuropsychiatric Interview version 5.0.0 (MINI) in a Norwegian translation was used for assessing Axis I disorders [57, 58].

### Personality disorders

The Structured Interview for DSM-IV (SIDP-IV) [59] in a Norwegian version was used to assess PDs. The SIDP-IV is a comprehensive semi-structured diagnostic interview for DSM-IV PD (Axis II) diagnoses. The SIDP-IV has been used in numerous studies in different countries, including Norway [60–62]. The SIDP-IV covers 14 DSM-IV Axis II diagnoses as well as CD as a separate axis I disorder. The Axis II diagnoses comprise the 10 standard DSM-IV PDs (paranoid, schizoid, schizotypal, borderline, histrionic, narcissistic, antisocial, obsessive-compulsive, dependent, and avoidant PD), the 3 provisional DSM-IV PDs (self-defeating, depressive, and negativistic PD), and mixed PD.

All questions address the typical or habitual behaviour of the subjects during the last 5 years. Each diagnostic criterion is rated on a four point scale: “0” = criterion not present; “1” = subthreshold level of the trait present; “2” = criterion being present for most of the last 5 years; and “3” = criterion strongly present. Scores “2” and “3” indicate the presence of a criterion according to DSM-IV [59].

In accordance with diagnostic practice applied in other studies on PDs in adolescence, the DSM-IV age criterion for ASPD was waived [17]. Due to the participants’ age, we also waived the 5 year symptom duration criterion. Instead we decided to use 2 years symptom duration as criterion. This is in accordance with the criterion used in previous studies assessing adolescent personality pathology [17, 18].

### Procedures and assessment

The first author assessed all participants. The parents or other legal guardians were not involved in the assessment process. The evaluator, male M.D., with 21 years of clinical experience, was specialist in psychiatry and child and adolescent psychiatry. He was trained in evaluation with SIDP-IV by the second author, who was an experienced rater, who had previously evaluated patients and reported from comparable studies [62, 63]. Twenty ratings were discussed and found to be in accordance with the rating of the experienced evaluator. ADHD and other Axis I conditions were also assessed by the same evaluator, who had been trained by the translator of the Norwegian version of the MINI.

After completion of the initial assessment, the patients were assigned to further clinical evaluation and treatment by clinicians other than the evaluator in the outpatient clinic.

### Statistical analysis

Descriptive statistics were calculated for the relevant mental health status variables and expressed in mean (SD) and frequency (%) as appropriate. Prevalences of ADHD, other Axis I conditions and PDs with 95 % Blaker confidence intervals [64] were estimated for the total sample and for each gender separately, with testing for gender differences by exact chi square tests. The total number of ADHD criteria and PD criteria was investigated graphically by locally weighted smoothing (lowess) curves. The relationship of PD with ADHD symptoms, unadjusted and adjusted for gender was investigated by logistic regression.

Adjustment for age and Axis I disorders was not performed due to the low number of degrees of freedom available. However, the relationship of the number of PD symptoms with ADHD symptoms, unadjusted and adjusted for gender, age and important Axis I disorders (simple phobias, generalized anxiety disorder, psychosis, major depressive episode, dysthymia, panic disorder, agoraphobia, social phobia, obsessive-compulsive disorder, post-traumatic stress disorder, CD, and abuse and dependency of alcohol and substances) was investigated by linear regressions wherein multicollinearity was checked by variance inflation factor (VIF), preferably below 5–10 for all covariates. Differences in unadjusted and adjusted odds ratios and regression coefficients were, when necessary, investigated by a bootstrap BC_a_ 95 % confidence intervals based on 10 000 bootstrap replicates [65], with a difference considered as significant if 0 was outside the interval.

Data were analysed using the IBM SPSS version 20.0 software, with Blaker confidence intervals computed in the R (The R Foundation for Statistical Computing, Vienna, Austria) package BlakerCI and bootstrapping in the R package boot. Graphical investigations also used R.

## Results

**Fig. 1:**
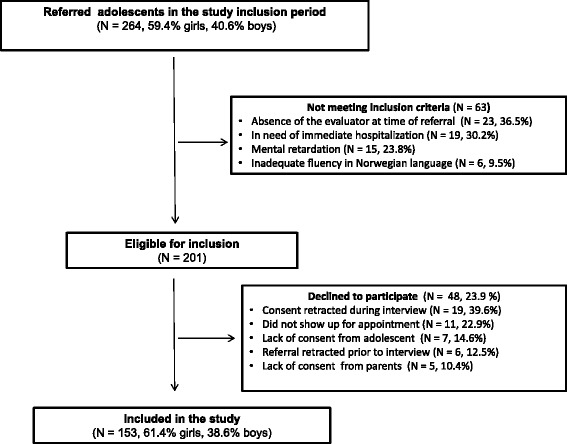
Flowchart of the patient recruitment and selection process

A total of 153 adolescents; mean age 16.0 years (SD = 1.1, minimum age 14.1 years, maximum age 18.0 years), 61.4 % (*N* = 94) girls were included in the study. There were no missing data on MINI and SIDP-IV. The flowchart in Fig. 1 illustrates the inclusion process and attrition.

Of the participants, 32.7 % (*N* = 50) initially screened positive for ADHD using the ASRS Screener. When using the MINI-PLUS as a diagnostic instrument, 13.7 % (*N* = 21, 95 % CI 8.9–20.1 %) of the adolescents fulfilled all diagnostic criteria for ADHD according to DSM-IV, with no significant gender difference in prevalence (Table 1).

**Table 1:** Prevalence of ADHD (*N* = 153)

ADHD	Boys (*N* = 59)	Girls (*N* = 94)	Total (*N* = 153)	*p*-value *
	N (%) (CI^a^)	N (%) (CI^a^)	N (%) (CI^a^)	
Without ADHD	50 (84.7 %) (73.2–92.0 %)	82 (87.2 %) (79.0–92.9 %)	132 (86.3 %) (79.9–91.0 %)	–
With ADHD	9 (15.3 %) (7.9–26.8 %)	12 (12.8 %) (7.1–21.0 %)	21 (13.7 %) (8.9–20.1 %)	0.810

^a^ Blaker 95 % confidence intervals

* *p*-value from exact chi square test

**Fig. 2:**
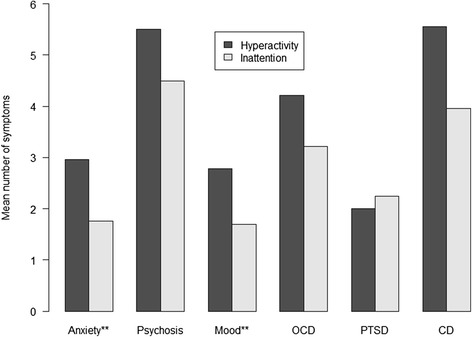
Frequency of hyperactivity/impulsiveness and inattention symptoms of ADHD by Axis I diagnosis*. * Anxiety: Anxiety disorders = Simple phobias, Generalized anxiety disorder, Panic disorder, Agoraphobia, Social phobia and Post-traumatic stress disorder; Psychosis: Psychotic disorders; Mood: Mood disorders = Dysthymia and Major depressive episode; OCD: Obsessive-compulsive disorder; PTSD: Post-traumatic stress disorder; CD: Conduct disorder ** p < 0.05

When analysed separately for hyperactivity/impulsiveness and inattention symptoms in each gender, girls had slightly higher overall symptom scores than boys, but the difference was not significant (hyperactivity; *χ*^2^ = 0.18, *p* = 0.786, inattention *χ*^2^ = 0.45, *p* = 0.668). The male/female ratio was 1.19 (95 % CI = 1.12–1.30). The distribution of hyperactivity/impulsiveness and inattention symptoms in different Axis I conditions can be seen in Fig. 2.

More than two thirds (68.6 %, *N* = 105) of the adolescents met the criteria for at least one Axis I disorder (76.6 %, *N* = 72 girls; 56.0 %, *N* = 33 boys). There were 16 boys (27.1 %) and 40 girls (42.6 %) with more than one Axis I disorder apart from ADHD (*p* = 0.060). Anxiety disorders; simple phobias, generalized anxiety disorder, panic disorder, agoraphobia, social phobia and post-traumatic stress disorder (33.3 %, *N* = 51, 95 % CI 26.0–41.1 %) and mood disorders; dysthymia and major depressive episode (32.7 %, *N* = 50, 95 % CI 25.3–40.5 %) were most frequent, followed by substance-related disorders; alcohol and drug abuse or dependence (18.3 %, *N* = 28, 95 % CI 12.6–25.3 %), CD (17.6 %, *N* = 27, 95 % CI 12.2–24.4 %), obsessive-compulsive disorder (9,2 %, *N* = 14, 95 % CI 5.3–14.8 %) and psychotic disorders (1.3 %, *N* = 2, 95 % CI 0.2–4.6 %). There were significant gender differences in anxiety (*p* = 0.022) and mood (*p* = 0.033) disorders. There were no bipolar, anorectic or bulimic patients in the sample (Table 2).

**Table 2:** Prevalence of Axis I disorders (*N* = 153)

Axis I disorders^b^	Boys (*N* = 59)	Girls (*N* = 94)	Total (*N* = 153)	*p*- value *
	N (%) (CI^a^)	N (%) (CI^a^)	N (%) (CI^a^)	
Anxiety	13 (22.0 %) (13.0–34.5 %)	38 (40.4 %) (30.7–50.7 %)	51 (33.3 %) (26.0–41.1 %)	0.022
Mood	13 (22.0 %) (13.0–34.5 %)	37 (39.4 %) (29.6–49.6)	50 (32.7 %) (25.3–40.5 %)	0.033
Psychosis	0 (0.0 %) (0.0–6.0 %)	2 (2.1 %) (0.4–7.1 %)	2 (1.3 %) (0.2–4.6 %)	0.523
OCD	4 (6.8 %) (2.3–16.4 %)	10 (10.6 %) (5.5–18.3 %)	14 (9.2 %) (5.3–14.8 %)	0.568
SUD	10 (16.9 %) (8.7–28.5 %)	18 (19.1 %) (11.9–28.5 %)	28 (18.3 %) (12.6–25.3 %)	0.831
CD	12 (20.3 %) (11.3–32.8 %)	15 (16.0 %) (9.5–24.8 %)	27 (17.6 %) (12.2–24.4 %)	0.519

^a^Blaker 95 % confidence intervals

^b^Axis I disorders: Anxiety = Anxiety disorders: Simple phobias, Generalized anxiety disorder, Panic disorder, Agoraphobia, Social phobia and Post-traumatic stress disorder. Mood = Mood disorders: Dysthymia and Major depressive episode. *OCD* Obsessive-compulsive disorder, *SUD* Substance-related disorders: Alcohol and drug abuse or dependence, *CD* Conduct disorder

*p-value from exact chi square test

Of the adolescents, 21.6 % (*N* = 33) had at least one PD, 7.2 % (*N* = 11) had more than one PD, and 4.6 % (*N* = 7) had both ADHD and a PD. The prevalence of PDs was generally higher in the referred girls. As shown in Table 3, no significant relationships between ADHD and specific PDs could be ascertained for boys. For girls, however, there were significant relationships between ADHD and ASPD (*p* = 0.002) and BPD (*p* = 0.042), as well as between ADHD and CD (*p* = 0.003). Only 3.4 % (*N* = 2) of boys and 3.2 % (*N* = 3) of girls, all with ADHD, matched the criteria for ASPD. There was no significant relationship with any other PDs (Table 3).

**Table 3:** Prevalence of specific personality disorders and conduct disorder in adolescents with ADHD (*N* = 153)

Personality Disorder (PD)	Boys with ADHD (*N* = 9)	Boys without ADHD (*N* = 50)	*χ* ^2^	*p*-value*	Girls with ADHD (*N* = 12)	Girls without ADHD (*N* = 82)	*χ* ^2^	*p*-value*
Paranoid	0	0	–	–	0	0	–	–
Schizoid	0	1	0.183	1.000	0	0	–	–
Schizotypal	0	0	–	–	0	0	–	–
Antisocial	1	1	–	–	3	0	21.176	0.002
Borderline	1	0	1.933	0.284	3	4	6.150	0.042
Histrionic	0	0	5.651	0.153	2	3	3.517	0.121
Narcissistic	0	0	–	–	0	1	0.148	1.000
Avoidant	0	3	0.569	1.000	0	6	0.938	0.598
Dependent	0	0	–	–	0	1	0.148	1.000
Obsessive-compulsive	0	0	–	–	0	6	0.938	0.598
Self-defeating	0	0	–	–	0	0	–	–
Depressive	0	2	0.373	1.000	0	8	1.280	0.383
Negativistic	0	0	–	–	1	1	2.544	0.240
At least one PD	1	7	0.054	1.000	6	19	3.860	0.076
More than one PD	1	0	5.651	0.153	2	8	0.526	0.611
Conduct disorder	3	8	1.107	0.369	6	8	11.887	0.003

*p-values from exact chi square tests

**Fig. 3:**
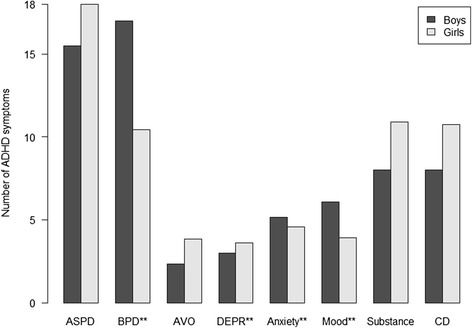
ADHD symptoms in adolescents with Axis I and personality disorders*. * ASPD = Antisocial personality disorder. BPD = Borderline personality disorder. AVO = Avoidant personality disorder. DEPR = Depressive personality disorder. Anxiety = Anxiety disorders; Simple phobias, Generalized anxiety disorder, Panic disorder, Agoraphobia, Social phobia and Post-traumatic stress disorder. Mood = Mood disorders; Dysthymia and Major depressive episode. Substance = Substance-related disorders; Alcohol and drug abuse and/or dependence. CD = Conduct disorder **p < 0.05

An illustration of the relationship between ADHD symptoms and relevant Axis I conditions and PDs is shown in Fig. 3. There were significant gender differences for BPD (*p* = 0.032), depressive PD (*p* = 0.020), anxiety disorders (*p* = 0.022), and mood disorders (*p* = 0.033). ASPD (*p* = 0.409), avoidant PD (*p* = 0.487), substance use disorders (*p* = 0.831), and CD (*p* = 0.585) did not yield significant gender differences.

There was no significant relationship between ADHD diagnosis and at least one PD, neither in unadjusted analysis (OR = 2.0, 95 % CI 0.7–5.6, *p* = 0.164) nor when adjusted for gender (OR = 2.2, 95 % CI 0.8–6.1, *p* = 0.138). No bootstrap procedure was considered necessary since these confidence intervals overlapped almost completely. Also, in unadjusted analysis the number of PD criteria was significantly higher (15.7, 95 % CI 6.3–25.1, *p* = 0.001) when ADHD diagnosis was present. In analysis adjusted for gender, age and Axis I disorders the corresponding estimate was 9.6, 95 % CI 1.2–18.0, *p* = 0.026. There was no significant difference between the unadjusted and adjusted estimate (95 % CI −0.52–13.43).

## Discussion

In the present study the prevalence of ADHD, common Axis I disorders, and gender differences were investigated in an unselected sample of adolescents. The participants were all referred to a non-specialized mental health outpatient clinic with a defined catchment area. We also investigated the relationship between ADHD and PD symptoms, as well as how this relationship was influenced by adjustment for Axis I disorders, age and gender.

We found that 13.7 % of the adolescents met the diagnostic criteria for ADHD. This was in accordance with previous findings, where studies of non-referred adolescents have found prevalence rates of 8.5 % [9], and prevalence rates in clinical samples are ranging from 11 to 16 % [39, 42]. When applying less strict diagnostic criteria than a definite DSM-IV diagnosis, prevalence rates in clinical samples of more than 30 % have been reported [43]. A similar discrepancy between screening and adherence to strict diagnostic criteria was found in the present study, in which 32.7 % of the adolescents screened positively for ADHD when using the ASRS Screener.

Earlier studies of ADHD have reported considerable prevalence differences between boys and girls [7, 8, 12]. In our material, however, there was no significant ADHD prevalence difference between the male and female adolescents. There was also no significant prevalence difference between genders when we analyzed hyperactivity/impulsiveness and inattention symptoms separately. This probably reflects that our sample was not preselected due to symptom severity or type, but the discrepancy is still considerable compared to the commonly assumed male/female ratio of 5:1 [12].

More than two thirds of the adolescents met the criteria for at least one Axis I disorder, with anxiety and mood disorders being most frequent. There were significant Axis I gender differences only in anxiety (*p* = 0.022) and mood (*p* = 0.033) disorders, with girls having the highest prevalence.

Previous studies have reported that the presence of a comorbid ADHD diagnosis influences the clinical presentation of BPD in adolescents [42]. The total prevalence of PDs in our material was 21.6 %, which was higher than previously reported from adolescent community samples and primary care settings [66], but lower than reported from selected, difficult-to-treat adolescent clinical and juvenile justice samples [18, 19, 67–70]. We found higher PD prevalences for girls, with ASPD and BPD reaching significant levels. All girls with ASPD also matched the diagnostic criteria for ADHD. This seems to be in accordance with studies of adults, where females with ADHD and BPD shared more clinical features than males [50, 51], and adult outpatients had a significant association between ADHD and BPD symptoms only in the female subsample [52].

Girls with ADHD were more severely ill than boys, with more Axis I and PD diagnoses. This may in part be explained by a selection bias due to only the most severely affected girls being referred to a mental health outpatient clinic. Also, in general clinical practice there may be more focus on assessing and diagnosing adolescent boys than girls presenting with ADHD symptoms, which suggests the possibility of an underestimation of the prevalence of ADHD in adolescent girls. One might speculate that boys are diagnosed with ADHD at a younger age, and that adolescent girls’ ADHD symptoms may be camouflaged by their PD symptoms.

The limited data size did not permit us to investigate the relationship between ADHD and single PDs. We did, however, find a significantly elevated number of PD symptoms in adolescents with an ADHD diagnosis (*p* = 0.001). When adjusted for age, gender and other Axis I disorders, this relationship was still significant (*p* = 0.026). Hence, the present study suggests that by using reliability-tested diagnostic interviews like the SIDP-IV, it is feasible to assess PDs in adolescents with ADHD, also in the presence of one or more comorbid Axis I disorders.

### Strengths and limitations

The study was performed at a single general service mental health outpatient clinic, receiving adolescents from a geographically defined urban area of varied socioeconomic and ethnic population. However, the results from the present study may not be generalizable to other populations. The attrition (23.9 %, *N* = 48) and the relatively small sample size constitute limitations. In particular, a limited number of degrees of freedom prevented the inclusion and investigation of interactions of potentially important adjustment variables like ADHD subtype. The participants were included in a limited time span, and we do not know if there were prevalence fluctuations over time.

The gender distribution of our sample was close to identical to the gender distribution of all referred adolescents in the study inclusion period, and reflects the fact that more adolescent girls than boys are referred to Norwegian mental health outpatient clinics.

Each patient was diagnosed individually with well-documented semi-structured interviews by a single, experienced clinician and rater. The MINI-PLUS, which utilizes the DSM-IV diagnostic criteria in a strict manner, was used for diagnosing ADHD. This was considered advantageous, as we did not want to overestimate the prevalence. The evaluator was trained in rating with SIDP-IV and MINI by experienced evaluators and researchers on PD and Axis I diagnoses. Still, the use of a single evaluator constitutes a possible limitation. This may have strengthened the internal validity, but might have been a threat to the external validity of the diagnoses.

## Conclusions

ADHD is an often lifelong condition with an extensive psychiatric comorbidity [2, 3].

It has been assumed that ADHD is 2 to 5 times more frequent in boys than in girls [7, 8]. We did, however, not find a significant gender difference with regard to the prevalence of ADHD in a typical sample of adolescents referred to a non-specialized mental health outpatient clinic. There was a significantly elevated number of PD symptoms in adolescents with an ADHD diagnosis, and this relationship did not significantly weaken when adjusted for age, gender and other Axis I disorders.

Girls with ADHD were more severely ill than boys with ADHD; we found higher PD prevalences for girls, with significant differences for ASPD and BPD. All girls with ASPD met the diagnostic criteria for ADHD.

The present study suggests that ADHD in girls in a general outpatient population may be more prevalent than previously assumed. It especially highlights the importance of assessing antisocial and borderline personality pathology in adolescent girls presenting with ADHD symptoms.

## Abbreviations

ADHD: attention deficit hyperactivity disorder
ASPD: antisocial personality disorder
BPD: borderline personality disorder
CD: conduct disorder
ODD: oppositional defiant disorder
PD: personality disorder
